# Regular Meeting of the Fulton County Medical Society, October 21, 1915

**Published:** 1915-10

**Authors:** Allen H. Bunce


### REGULAR MEETING OF THE FULTON COUNTY MEDICAL SOCIETY, OCTOBER 21, 1915.

By Dr. Allen H. Bunce.

Dr. John Funke discussed: "The Amoeba, its Etiological Relationship to Pyorrhea Alveolaris."

Dr. E. C. Thrash in discussing the subject stated that he had been able to demonstrate the amoeba in every case of pyorrhea he had examined. He does not know of how much importance the amoeba is in the causation of the pyorrhea, but is certain of the fact of their presence. For local application to the gums he does not consider emetine superior to iodine. The surgical procedures of tartar and the deposit of calculus on the teeth and the diseased peridental membrane make pyorrhea a surgical condition just as appendicitis is a surgical condition. This surgical work must be done by the dental surgeon and done well before any other local or systemic treatment will be of benefit.

Dr. Edgar Paullin stated that he was not sure what part the amoeba played in the causation of pyorrhea, especially since the Dental Research Commission has advanced the theory that amoebae cure the disease rather than cause it. In a large number of examinations of pyorrhea mouths Dr. Paullin has found the amoeba in practically all cases, especially so in acute cases. He has found it necessary at times to make several examinations before being able to demonstrate the parasite. He has found that amoebae can be more easily demonstrated in a fresh specimen than in the dry state. In order to cure the disease the calculus formation and diseased bone must be removed by a dental surgeon before any other procedures are undertaken. He doubts that cases where destruction of bone has taken place ever become cured. He finds that individuals invariably relapse unless prophylaphtic measures are kept up.

Dr. Claude Smith reported that in his experience in examining the flora of the mouth for the last fifteen years that he had found the amoeba present in almost all mouths. Many other organisms are constantly found present in the mouth. In examining for amoeba he found it necessary to make repeated examinations and he considers one negative examination of very little value. In his experience fresh specimens are much superior for the demonstration of these parasites. There are other conditions both local and general which contribute to the return of pyorrheal conditions after the patient has once been cured. Quite often oral conditions are a result and not a primary cause of the trouble we find present.

Dr. E. C. Cartledge reported that amoebae had been found in the normal mouth. He also observed a case in which spontaneous cure took place and asked if there was any explanation to this fact.

Dr. Funke in closing said that cases of pyorrhea are cured but that once having had pyorrhea renders the patient more susceptible to a return of the disease. He is also positive of the fact that in some cases of pyorrhea the amoeba is not present and where the amoeba is not present the use of emetine is of little or no value. In those cases where amoebae are not present the disease does not clear up so rapidly as where they are present. The tartar must be removed thoroughly before any local or general treatment is undertaken.

Dr. G. D. Ayer read a paper on: "Vernal Catarrh."

In discussing this subject Dr. R. R. Daly remarked that vernal catarrh is a rare disease in this locality. In his clinics he has found it only occasionally during a period of several years, although he has watched for the condition carefully. In his experience he has found that the disease lasts for a long period of time and is very difficult to cure. The pain and photophobia are the most difficult symptoms with which he has had to contend. He commended Dr. Ayer very highly for finding a remedy which will lessen the duration and decrease the discomfort of the disease.

Dr. Howard Hall doubts the correct diagnosis of the case reported by Dr. Ayer. In his experience he has found no discharge in vernal catarrh. He finds no puffing of the lids and has never seen a case cured so easily. In his opinion the cases reported cured in a short time are cases in which the diagnosis was incorrect.

Dr. R. M. Nelson has seen a number of cases in his clinic at the Atlanta Medical College and he has noted that the bulbar conjunctiva is especially affected. Quite often he has noticed the conjunctiva of the upper lid to be the seat of the most trouble. In his opinion it is better to advise a change of climate for these cases as the disease often lasts for eight or ten years. He advises further observation on the remedy advocated by Dr. Ayer.

Dr. E. C. Thrash asked for the difference between vernal catarrh and hay fever.

Dr. Gerald Selby who made the examinations in the case reported by Dr. Ayer stated that the discharge in this case was not of a purulent nature. There was only a small amount of discharge and this consisted of a thin serous substance. He reported finding certain bodies which have been previously described as pathognomonic of vernal catarrh. These bodies were present in this case in considerable numbers. (A description of them will be seen in Dr. Ayer's article). In making a study of the cellular contents of this discharge Dr. Selby found a predominance of eosinophiles which is always characteristic of this disease. There was considerable edema of the conjunctiva at the time of Dr. Selby's examination. After the patient had been treated Dr. Selby made a second examination in which he found none of the bodies as reported above and no eosinophiles.

In closing Dr. Ayer remarked that he had noticed a prompt improvement in the condition of his patient immediately after the injection of the emetine hydrochloride. He made a careful study of the case and was sure of the diagnosis before he instituted treatment. The examination of the secretions was made before any antiseptics were used.

---

A suction apparatus is a most desirable addition to the surgeon's armamentarium for the cleanly emptying of a distended gall-bladder and for the quick removal of accumulations in the abdomen of blood (ectopic pregnancy, rupture of the liver, etc.) or pus (purulent peritonitis, pelvic abscess, etc.). It removes these fluids without disseminating them and, since the suction is continued while the operation proceeds, it obviates endless sponging and saves an amount of time that, in some of these cases, may be the margin between recovery and death.

